# Critical appraisal of irrational drug combinations: A call for awareness in undergraduate medical students

**DOI:** 10.4103/0976-500X.77117

**Published:** 2011

**Authors:** Shilpa P Jadav, Dinesh M Parmar

**Affiliations:** *Department of Pharmacology, M.P. Shah Medical College, Jamnagar 361 008, India*

Dear Editor,

The conference of experts on the rational use of drugs, convened by the World Health Organization (WHO) in Nairobi in 1985, stated that *Rational use of drugs requires that patients receive medications appropriate to their clinical needs, in doses that meet their own individual requirements for an adequate period of time, at the lowest cost to them and their community.* Irrational or non-rational use is the use of medicines in a way that is not compliant with rational use as defined above. The use of too many medicines per patient known as polypharmacy is one of the common types of irrational medicine use.[1]

Formulations containing two or more drugs in combination in a fixed ratio are called fixed dose combinations (FDCs). Drugs having the same effect of action are known as homergic drugs while two drugs are said to be heterergic if they do not cause response of the same quality. Homergic drug combinations have advantages of reduced dose and increase in therapeutic action (e.g., amlodipine + enalapril) while heterergic drug combinations have advantages of decreased untoward reaction (e.g., ramipril + hydrochlorothiazide), prolonged duration of action (e.g., probenecid + penicillin), modified absorption (e.g., lignocaine + adrenaline), or decreased metabolism (e.g., levodopa + carbidopa).[2]

## Advantages of fixed dose combinations[3]

1. Combination medicines have the advantages of combination therapy as well as advantages related to reducing the number of pills to be taken.
2. Reducing the number of pills diminishes the complexity of the regimen and therefore leads to improved patient adherence.
3. Reduced administration costs stem from simplified packaging, fewer prescriptions, and lesser dispensing time and cost.
4. FDCs can improve compliance in the treatment of chronic infectious disease, where partial adherence can lead to the development of drug-resistant strains, treatment failure, and a threat to public health, e.g., treatment of TB and HIV.
5. The side effects of one medicine can be reduced by combining it with another medicine in a FDCs, e.g., levodopa + carbidopa.
6. The efficacy of one medicine can be synergistically increased by combining it with another, e.g., estrogen + progesterone, sulfamethoxazole + trimethoprim.

## Disadvantages of fixed dose combinations[3]

1. Titration of dose of medicine to suit individual patients is not possible, e.g., atorvastatin 10 mg + amlodipine 5 mg.
2. FDCs increase the price of the medication if unnecessary drugs are included, e.g., ibuprofen + paracetamol + caffeine.
3. One of the drugs in the combination may be superfluous or wasteful, e.g., vitamins + iron.
4. The incidence of adverse effects increases, e.g., nimesulide + paracetamol.
5. In FDCs, there is always a chance that individual medicines may not be present in adequate amounts, e.g., multivitamins.
6. Incompatible pharmacodynamics, e.g., combination of an antihistaminic with an antidiarrheal is dangerous as the antihistaminic action may mask other symptoms and make accurate diagnosis and treatment difficult.
7. It is difficult to identify which medicine in the FDCs has caused an adverse effect.

The 16^th^ essential medicines list of WHO have 351 essential medicines, including 26 FDCs[4] [Table 1], whereas the national list of essential medicines of India have 354 essential drugs, including 14 drug combinations[5] [Table 2]. Despite of this information over 70 dangerous FDCs are being sold in India under more than 1000 brand names,[6] some of them are categorized here.

- FDCs of cardiovascular drugs such as ramipril + telmisartan are associated with more adverse events without offering any increase in benefits.[7]
- FDCs of analgesic, anti-inflammatory and antipyretic like nimesulide + paracetamol having increased hepatotoxic adverse effects.[8]
- FDCs of hypolipidemic drugs such as atorvastatin + nicotinic acid combination having increased probability of myopathy.[8]
- FDCs of gastrointestinal drugs such as domperidone + rabeprazole having increased incidence of rhabdomyolysis.[8]
- FDCs of cough and cold remedies such as cetirizine + phenylpropanolamine + dextromethorphan; in this combination phenylpropanolamine is a banned drug which has potential to cause stroke.[8]
- FDCs of antimicrobials such as fluconazole + tinidazole are irrational because the patient may need only one drug after making a correct diagnosis.[8]
- FDCs of antimicrobials such as amoxycillin + cloxacillin; in this combination, amoxycillin is inactive against staphylococcus as most strains produce β-lactamase and cloxacillin is not so active against streptococci. Therefore, for any given infection, one of the components is useless and adds to cost and adverse effects. Further, since the amount of each drug is halved, efficacy is reduced and chances of selecting resistant strains are increased.[8]

**Table 1:** Fixed dose combinations included in 16^th^ WHO model list of essential medicines

Drug combinations
Amoxicillin + Clavulanic acid
Efavirenz + Emtricitabine + Tenofovir
Emtricitabine + Tenofovir
Lamivudine + Nevirapine + Stavudine
Lamivudine + Nevirapine + Zidovudine
Lopinavir + Ritonavir
Lamivudine + Zidovudine
Ethambutol + Isoniazid
Ethambutol + Isoniazid + Pyrazinamide + Rifampicin
Ethambutol + Isoniazid + Rifampicin
Isoniazid + Pyrazinamide + Rifampicin
Isoniazid + Rifampicin
Artemether + Lumefantrine
Sulfadoxine + Pyrimethamine
Sulfamethoxazole + Trimethoprim (Oral)
Sulfamethoxazole + Trimethoprim (Injection)
Neomycin sulfate + Bacitracin
Imipenem + Cilastatin
Ethinylestradiol + Levonorgestrel
Ethinylestradiol + Norethisterone
Estradiol cypionate + Medroxyprogesterone acetate
Levodopa + Carbidopa
Ferrous salt + Folic acid
Lidocaine + Epinephrine (Adrenaline)
Benzoic acid + Salicylic acid
Oral rehydration salts (sodium chloride, trisodium citrate dehydrate, potassium chloride, glucose)

**Table 2:** Fixed dose combinations included in national essential medicine list of India

Drug combinations
Co-trimoxazole (Trimethoprim + Sulfamethoxazole)
Lamivudine + Nevirapine + Stavudine)
Lamivudine + Zidovudine
Thiacetazone + Isoniazid
Sulfadoxine + Pyrimethamine
Neomycin + Bacitracin
Ethinylestradiol + Levonorgesterol
Ethinylestradiol + Norethisterone
Levodopa + Carbidopa
Lignocaine hydrochloride + Adrenaline
Acriflavin + Glycerin
Benzoic acid + Salicylic acid
Aluminum hydroxide + Magnesium hydroxide
Oral rehydration salts (sodium chloride, trisodium citrate dehydrate, potassium chloride, glucose)

The above-mentioned magnitude of the problem of prescribing irrational FDCs can be reduced by emphasizing the principles of rational drug use selection such as efficacy, safety, suitability, and cost effectiveness in each given case. Since the medical undergraduates, later in their profession will have to play an important role in avoiding irrational FDCs, an effective undergraduate training is necessary in rational prescribing process. Hence, the concept of critical appraisal of irrational drug combinations in undergraduate pharmacology practical (UGPP) curriculum is emphasized through this correspondence.

Many doctors, both in government as well as private, prescribe irrational FDCs using the excuse of better patient compliance.[8] Diarrheal diseases (e.g., amoebic or bacterial) are commonly encountered in clinical practice which are being treated with FDCs of antimicrobial agents. Thus, we have decided to choose diarrhea and its treatment as an example in this article.

Many patients with sudden-onset of diarrhea have a benign, self-limited illness requiring no treatment, but in severe cases oral rehydration therapy is a cornerstone as dehydration and electrolyte imbalances are the principle risk.[9] Antimicrobial drugs have a limited role in the treatment of diarrheal patients because even in bacterial diarrhea they alter the course of illness only in selected cases and may prolong the carrier state.[10] FDCs of an antiprotozoal and an antibacterial, for the treatment of diarrhea, have been available in the Indian pharmaceutical market for about a decade.[11] In this paper, we will discuss one of such FDCs prescribed by doctors in the treatment of diarrhea (e.g., norfloxacin + metronidazole) to understand its irrationality. After a discussion on FDCs as described earlier in this article, a prescription containing irrational FDCs for a particular condition is to be given to a group of four to five students for critical appraisal using rationality criteria [refer Exercises 1(a) and 1(b) given below]. At the end of the practical class, the student should be able to:

1. Understand the concept of FDCs in terms of definition, advantages, and disadvantages of FDCs.
2. Differentiate the rational from irrational FDCs.
3. Critically analyze the irrational FDCs in terms of efficacy, safety, and cost effectiveness of FDCs given in a prescription.

## Exercise 1 (a)

A doctor has diagnosed *acute amoebic dysentery* in an adult patient and prescribed a combination of norfloxacin 400 mg + metronidazole 500 mg to be taken orally twice daily for 5 days. Analyze the given combination using rationality criteria.

## Solution

To define the diagnosis is the first step in the process of choosing a P (personal) drug.[12] There is a definite diagnosis for this case, and if it is the most appropriate one should start the definite drug treatment for that condition. Therefore, this case should be treated with metronidazole followed by diloxanide furoate [see later].

## Justification

A number of combinations of nitroimidazole (antiamoebic) with fluoroquinolone (antibacterial) available in the Indian market are irrational because patient suffers only from one type of diarrhea and therefore use of such a combination adds to the cost, adverse effects, and may encourage resistance.[8]

### Efficacy

FDCs should be avoided when one drug is adequate for the said condition. Metronidazole is effective in acute amoebic dysentery and no need to give norfloxacin. Therefore, metronidazole followed by diloxanide furoate to get rid of the cysts that may remain after the metronidazole therapy is the treatment part of this case.[13]

### Safety

By using norfloxacin + metronidazole combination, adverse effects commonly associated with norfloxacin (fluoroquinolone) such as mild nausea, vomiting, and abdominal discomfort would be added to adverse effects of metronidazole (see later).

### Cost effectiveness

Rational therapy calls for the prescription of less-costly single ingredient drugs more often than costlier combination agents.[11] Metronidazole is to be administered as 750 mg three times a day for 5 days[13] while the combination of norfloxacin 400 mg + metronidazole 500 mg is given twice daily for 5 days in acute amoebic dysentery. Average minimum cost for 10 tablets of metronidazole 400 mg is Rs. 03.10 and so the cost of 30 tablets of the same strength would be about Rs. 10.00. On the other hand, average minimum cost for 10 tablets of combination of norfloxacin 400 mg + metronidazole 500 mg is Rs. 45.00.[14] Thus, the average minimal cost of therapy of acute amoebic dysentery with metronidazole and with combination of norfloxacin + metronidazole would be Rs. 10.00 and Rs. 45.00, respectively. This shows difference of Rs. 35.00 which clearly indicates that the combination of norfloxacin + metronidazole is more expensive than metronidazole.

## Exercise 1(b)

A doctor has diagnosed *acute severe bacterial dysentery* in an adult patient and prescribed a combination of norfloxacin 400 mg + metronidazole 500 mg to be taken orally twice daily for 5 days. Analyze this combination using rationality criteria.

## Solution

Again there is a definite diagnosis in this case also and therefore considering the P-drug concept, norfloxacin should be prescribed for this case.

## Justification

It will remain the same as discussed in Exercise 1 (a).

### Efficacy

Antimicrobials are useful only in severe cases generally caused by enteroinvasive organisms such as *Shigella, Enteropatogenic E. coli (EPEC), Campylobacter jejuni, Salmonella typhimurium, and Yersinia enterocolitica*. Antimicrobials such as co-trimoxazole, norfloxacin, doxycycline, and erythromycin are needed in such cases.[10] Norfloxacin is still the most cost-effective choice for bacterial diarrhea in adults.[11] Further, for adults with acute diarrhea, there is good evidence that an ultrashort course (one or two doses) of ciprofloxacin or another fluoroquinolone reduces the severity and shortens the duration of acute traveler’s diarrhea.[13] Therefore, only norfloxacin should be prescribed in this case.

### Safety

Most common side effects such as headache, nausea, dry mouth, and metallic taste while occasional side effects such as vomiting, diarrhea, and abdominal distress associated with metronidazole would be added to adverse effects of norfloxacin (see above).

### Cost effectiveness

Norfloxacin is to be administered as 400 mg two times a day for 3 days[13] while the combination of norfloxacin 400 mg + metronidazole 500 mg is given as twice daily for 5 days in acute bacterial dysentery. Average minimum cost for 10 tablets of norfloxacin 400 mg is Rs. 20.00, so the cost of 6 tablets of the same strength would be about Rs. 12.00. On the other hand, average minimum cost for 10 tablets of combination of norfloxacin 400 mg + metronidazole 500 mg is Rs. 45.00.[14] Therefore, the average minimal cost of therapy of acute severe bacterial dysentery with norfloxacin and with combination of norfloxacin + metronidazole would be Rs. 12.00 and Rs. 45.00, respectively. This shows difference of Rs. 33.00 which indicates that the combination of norfloxacin + metronidazole is more expensive than norfloxacin.

To sum up, the study of Exercises 1(a) and 1(b) indicates that the use of irrational FDCs adds unnecessary cost and adverse effects to the therapy. In addition, injudicious use of antimicrobial agents may lead to the development of resistant organisms too. Hence, undergraduate medical students should be sensitized to this fact by inclusion of such exercises in UGPP curriculum.
